# Cardiorenal Effects of Switching from Eplerenone to Esaxerenone in Patients with Chronic Heart Failure and Hypertension: A Prospective Clinical Study

**DOI:** 10.3390/jpm16070388

**Published:** 2026-07-20

**Authors:** Akira Sezai, Masanori Abe, Takashi Maruyama, Makoto Taoka, Hisakuni Sekino, Masashi Tanaka

**Affiliations:** 1 Department of Cardiovascular Surgery, Nihon University School of Medicine, 30-1 Ohyaguchi-Kamicho, Itabashi-Ku, Tokyo 173-8610, Japan; taoka.makoto@nihon-u.ac.jp (M.T.); tanaka.masashi@nihon-u.ac.jp (M.T.); 2 Division of Nephrology, Hypertension and Endocrinology, Department of Medicine, Nihon University School of Medicine, Tokyo 173-8610, Japan; abe.masanori@nihon-u.ac.jp (M.A.); maruyama.takashi@nihon-u.ac.jp (T.M.); 3 Sekino Hospital, Tokyo 171-0014, Japan; sekinoh@sekino-hospital.com

**Keywords:** heart failure, hypertension, mineralocorticoid receptor antagonist, esaxerenone, cardiorenal syndrome

## Abstract

**Background/Objectives:** Esaxerenone is a non-steroidal mineralocorticoid receptor antagonist (MRA) with potent cardiorenal protective effects. However, the clinical effects of switching from eplerenone to esaxerenone in patients with chronic heart failure complicated by hypertension remain unclear. This study investigated the effects of switching from eplerenone to esaxerenone on blood pressure, heart failure biomarkers, renal function, and the renin–angiotensin–aldosterone system (RAAS). **Methods:** A total of 156 patients with chronic heart failure and hypertension who had been receiving eplerenone for more than one year were prospectively enrolled. Eplerenone was switched to esaxerenone, and the patients were followed for 6 months. Blood pressure, heart rate, brain natriuretic peptide (BNP), renal function, urinary albumin-to-creatinine ratio (UACR), plasma renin activity (PRA), plasma aldosterone concentration (PAC), and urinary osmolality (U-OSM) were evaluated. **Results:** Following the switch to esaxerenone, systolic and diastolic blood pressure significantly decreased (both *p* < 0.001), whereas heart rate remained unchanged. BNP levels significantly decreased at 3 and 6 months (*p* = 0.008 and *p* = 0.002, respectively). Serum creatinine decreased (*p* = 0.017), estimated glomerular filtration rate increased (*p* = 0.028), and UACR significantly decreased at both time points (both *p* < 0.001). PRA and PAC significantly increased after switching (both *p* < 0.05), whereas angiotensin II levels remained unchanged. U-OSM significantly decreased (*p* = 0.01 and *p* = 0.002 at 3 and 6 months, respectively). No major cardiovascular events or severe adverse events were observed. **Conclusions:** In patients with chronic heart failure and hypertension, switching from eplerenone to esaxerenone was associated with reductions in blood pressure, BNP, UACR, and urinary osmolality, together with improvements in renal function. These findings suggest favorable physiological changes following the switch from a steroidal to a non-steroidal mineralocorticoid receptor antagonist, although confirmation in randomized controlled studies with clinical outcome measures is warranted.

## 1. Introduction

Mineralocorticoid receptor antagonists (MRAs) are important medications for the treatment of heart failure and hypertension. Spironolactone, a first-generation MRA, has been shown in numerous large-scale studies to be effective against heart failure and hypertension [1,2]. However, it has been associated with hormonal side effects, such as gynecomastia. In the RALES trial, which enrolled heart failure patients with a left ventricular ejection fraction (EF) of less than 35%, spironolactone reduced all-cause mortality, heart failure-related mortality, and hospitalizations due to heart failure [1]. Eplerenone, a second-generation MRA, has demonstrated efficacy in patients with heart failure in several clinical trials. Furthermore, because of its high selectivity for the mineralocorticoid receptor (MR), it is considered less likely to cause hormonal side effects. In the EPHESUS trial, which enrolled patients with acute myocardial infarction and an EF of 40% or less, eplerenone reduced all-cause mortality, cardiovascular mortality, and hospitalizations for heart failure [3]. In the EMPHASIS-HF trial, which enrolled patients with chronic heart failure and an EF of 35% or less, eplerenone also reduced all-cause mortality, cardiovascular mortality, and heart failure hospitalizations [4]. Spironolactone and eplerenone are MRAs with a steroid skeleton. In recent years, the non-steroidal MRAs esaxerenone and finerenone have emerged and are classified as third-generation MRAs. In the FIDELIO-DKD and FIGARO-DKD trials, which focused on chronic kidney disease (CKD) patients with type 2 diabetes, finerenone reduced renal composite events and cardiovascular composite events [5,6]. Subsequently, in the FINEARTS-HF trial, which included heart failure patients with an EF of 40% or higher, finerenone reduced the composite of total heart failure worsening events and cardiovascular death [7]. Esaxerenone is an MRA developed in Japan. Esaxerenone is less likely to cause hormonal side effects because of its high selectivity for the mineralocorticoid receptor and its non-steroidal structure [8,9,10] (Table 1). Although esaxerenone is approved for the treatment of hypertension, it has not been approved for heart failure, and its efficacy in patients with chronic heart failure remains unclear.

The authors previously reported in a crossover trial involving patients with chronic heart failure and hypertension that esaxerenone improved brain natriuretic peptide (BNP), blood pressure, and urinary albumin/creatinine ratio (UACR) compared with eplerenone, while also increasing plasma aldosterone concentration (PAC) [11]. However, it remains unclear whether these findings can be replicated in routine clinical practice after switching from a steroidal to a non-steroidal mineralocorticoid receptor antagonist. Therefore, in this study, we investigated the effects of switching from eplerenone to esaxerenone on blood pressure, heart failure-related biomarkers, renal function, and the renin–angiotensin–aldosterone system in patients with chronic heart failure and hypertension. We hypothesized that switching from eplerenone to esaxerenone would be associated with favorable cardiorenal changes despite compensatory activation of the RAAS.

## 2. Materials and Methods

### 2.1. Study Protocol

This study enrolled patients with chronic heart failure complicated by hypertension who had been taking eplerenone for more than one year. Eplerenone (Serara^®^, Viatris Inc., Tokyo, Japan) was switched to esaxerenone (Mineburo^®^, Daiichi Sankyo Company, Limited, Tokyo, Japan). Patients taking 25 mg/day of eplerenone were switched to 1.25 mg/day of esaxerenone, with the dose increased to 2.5 mg/day the following month; patients taking 50 mg/day of eplerenone were switched to 2.5 mg/day of esaxerenone, with the dose increased to 5 mg/day the following month, and both groups continued treatment for 6 months. The dose conversion strategy was based on the approved Japanese prescribing information for esaxerenone and previous clinical evidence, including the ESAX-HTN study.

Eligible patients had chronic heart failure and hypertension treated with eplerenone and one or more additional antihypertensive agents. Chronic heart failure was defined as a history of hospitalization for heart failure and treatment with two or more standard heart failure medications (angiotensin-converting enzyme inhibitors, angiotensin II receptor blockers, angiotensin receptor–neprilysin inhibitors, β-blockers, diuretics, sodium–glucose cotransporter 2 inhibitors, or inotropic agents). NYHA functional class and left ventricular ejection fraction are summarized in Table 2. Eligible patients were 20–89 years of age. Patients were excluded if they met either of the following criteria: (1) an estimated glomerular filtration rate < 30 mL/min/1.73 m^2^ or (2) were considered unsuitable for participation by the attending physician. No washout period was introduced because temporary discontinuation of mineralocorticoid receptor antagonist therapy was considered ethically inappropriate, as it could increase the risk of worsening heart failure or loss of blood pressure control. All consecutive eligible patients during the study period were enrolled; therefore, no formal sample size calculation was performed.

Blood pressure was assessed using early-morning home blood pressure measurements throughout the study. If systolic blood pressure was 140 mmHg or higher after initiation of esaxerenone, calcium channel blockers or alpha-blockers—which have minimal effects on the RAAS—were added or their doses were increased. If systolic blood pressure fell to 90 mmHg or lower after initiation of esaxerenone, or if symptoms such as dizziness were observed, calcium channel blockers or alpha-blockers were discontinued or their doses were reduced in patients receiving these agents.

The details of the study were explained to each patient and written informed consent was obtained from all participants. The study was approved by the hospital’s institutional review board (protocol no. 20181201-2; approval on 2 April 2019), and the study was registered with the University Hospital Medical Information Network (UMIN) Clinical Trials Registry (study ID: UMIN000037113).

Endpoints: The primary endpoints were home morning blood pressure and heart rate. The secondary endpoints were as follows: (1) BNP and atrial natriuretic peptide (ANP), (2) blood urea nitrogen (BUN), serum creatinine (s-Cr), estimated glomerular filtration rate (eGFR), (3) plasma renin activity (PRA), angiotensin II (Ang II), PAC, cortisol, (4) sodium, potassium (serum, urinary), urinary sodium-to-potassium ratio (U-Na/K), osmolality (OSM) (serum, urinary), (5) UACR, urinary type IV collagen (U-IV collagen), urinary β_2_-microglobulin (U-β_2_MG), urinary liver-type fatty acid-binding protein (U-LFABP). Blood samples were collected for all parameters prior to initiation of esaxerenone. Following initiation of esaxerenone, BNP, ANP, BUN, sCr, eGFR, serum sodium, and potassium were measured at 1, 3, and 6 months post-administration, while PRA, Ang II, PAC, cortisol, UACR, U-IV collagen, U-β_2_MG, and U-LFABP were measured at 3 and 6 months post-administration.

Adverse reactions were classified as renal dysfunction (≥50% increase in serum creatinine), hepatic dysfunction (≥50% increase in AST or ALT), and allergic reactions. The management of adverse reactions was determined by the attending physician. Major adverse cardiovascular events were defined as death or hospitalization due to ischemic heart disease, cerebrovascular disease, heart failure, or arrhythmia.

### 2.2. Statistical Analysis

Continuous variables are expressed as mean ± standard deviation for normally distributed data and as median (interquartile range) for non-normally distributed data. Variables with skewed distributions (e.g., BNP, ANP, and RAAS markers) are log-transformed prior to analysis.

Longitudinal changes over time were analyzed using linear mixed-effects models with time as a fixed effect and a random intercept for each subject. An autoregressive covariance structure of order 1 [AR(1)] was used to account for within-subject correlations across repeated measurements. Estimated marginal means are presented with standard errors. Post hoc pairwise comparisons between baseline and each follow-up time point were adjusted using the Bonferroni correction. A two-sided *p*-value < 0.05 was considered statistically significant. All analyses were performed with SPSS software (version 28.0.0.0; IBM Corp., Armonk, NY, USA). Data aggregation was performed by Sekino Laboratory staff who were not involved in this study.

## 3. Results

A total of 156 patients were enrolled in this study. The baseline characteristics are shown in Table 1. Regarding heart failure classifications, heart failure with reduced ejection fraction (HFrEF) accounted for 7%, heart failure with mildly reduced ejection fraction (HFmrEF) for 5%, and heart failure with preserved ejection fraction (HFpEF) for 88%; thus, the majority of patients enrolled in this trial had HFpEF. Among patients with CKD, 71% were classified as CKD stage G3a and 29% as CKD stage G3b (Table 2).

Adverse Events

**Figure 1:**
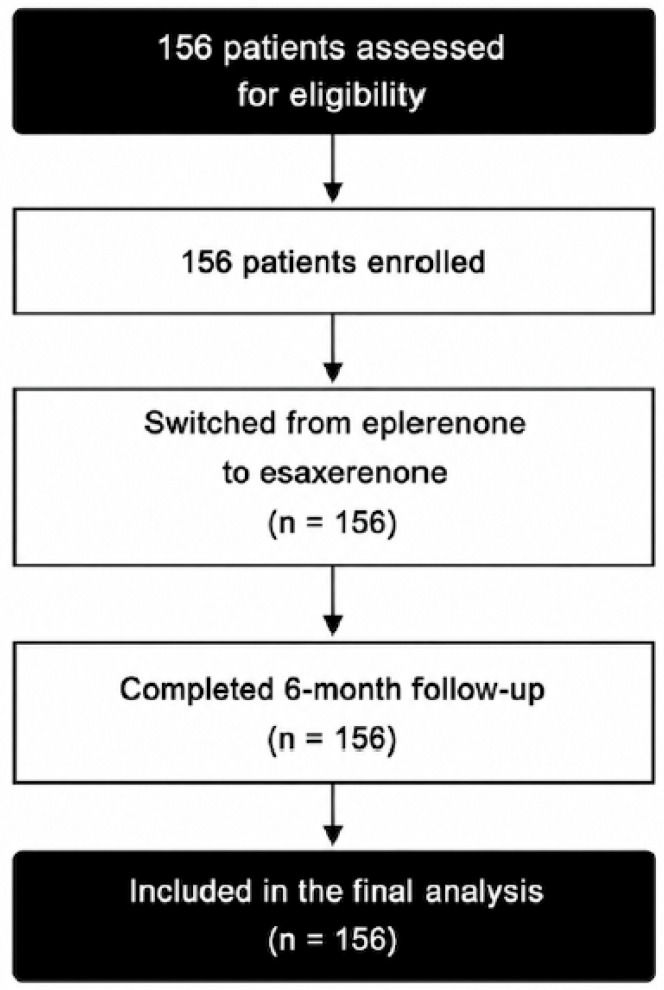
Flow diagram of the study.

During follow-up, no patient required intensification of antihypertensive therapy because systolic blood pressure ≥ 140 mmHg was not observed. In contrast, one patient developed systolic blood pressure < 90 mmHg at 1 month after switching, and two patients experienced dizziness at 3 months, resulting in a reduction in the dose of a calcium channel blocker. There was no worsening of heart failure, and no cardiovascular events occurred. No patients discontinued treatment because of liver dysfunction, renal dysfunction, hyperkalemia, or gynecomastia, and all patients completed the 6-month follow-up. (Figure 1).

**Figure 2:**
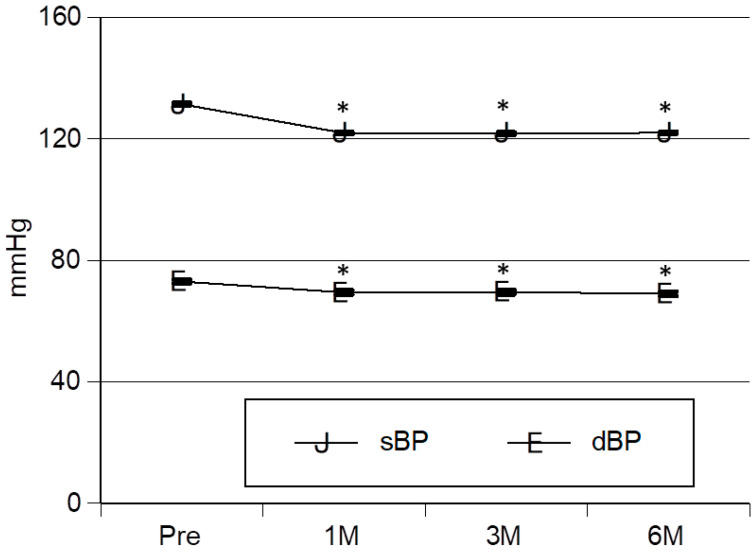
Changes in systolic and diastolic blood pressure after switching from eplerenone to esaxerenone. Values are expressed as mean ± SEM. * *p* < 0.05 vs. Pre.

Primary endpoints:

Blood pressure and heart rate (Figure 2 and Table 3): Regarding systolic blood pressure, it was 131.5 ± 12.4 mmHg during eplerenone treatment; one month after switching to esaxerenone, it was 121.9 ± 9.9 mmHg; three months after switching, it was 121.8 ± 9.8 mmHg; and 122.0 ± 9.5 mmHg at 6 months. For diastolic blood pressure, it was 73.0 ± 10.3 mmHg during eplerenone treatment, 69.4 ± 8.5 mmHg one month after switching to esaxerenone, 69.5 ± 8.5 mmHg at three months, and 69.0 ± 10.3 mmHg at six months. Both systolic and diastolic blood pressure decreased significantly after switching from eplerenone to esaxerenone (all *p* < 0.001). Heart rate was 66.4 ± 8.8 beats per minute during eplerenone treatment; one month after switching to esaxerenone, it was 65.4 ± 8.5 beats per minute; at three months, 66.0 ± 8.9 beats per minute; and at six months, 65.4 ± 8.0 beats per minute, with no significant difference (*p* = 0.215).

**Figure 3:**
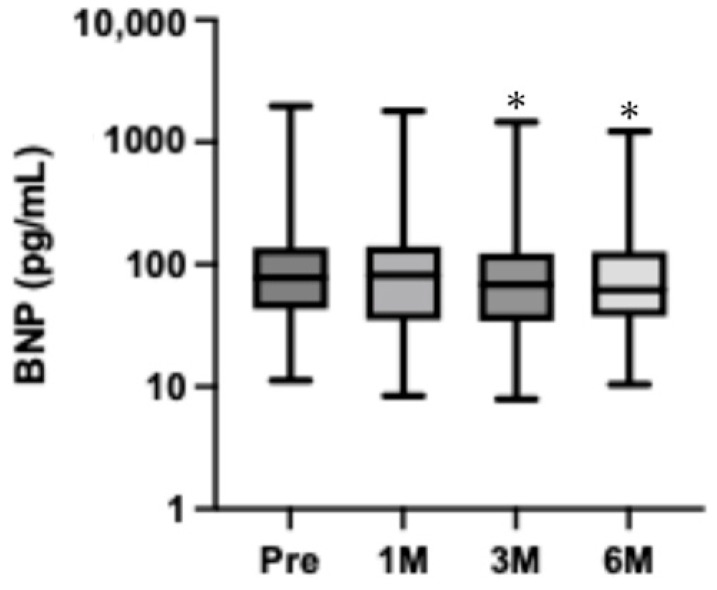
Changes in BNP after switching from eplerenone to esaxerenone. BNP, brain natriuretic peptide. * *p* < 0.05 vs. Pre.

**Figure 4:**
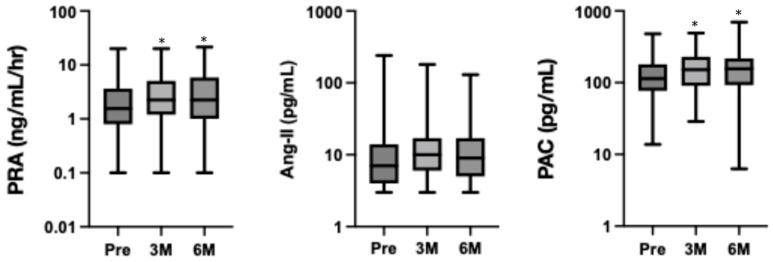
Changes in PRA, Ang-II, and PAC after switching from eplerenone to esaxerenone. PRA, plasma renin activity; Ang-II, angiotensin II; PAC, plasma aldosterone concentration. * *p* < 0.05 vs. Pre.

**Figure 5:**
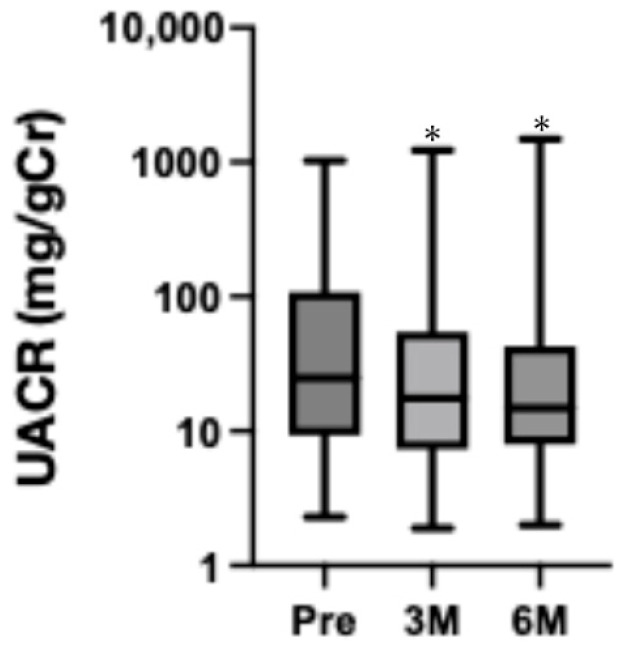
Changes in UACR after switching from eplerenone to esaxerenone. UACR, urinary albumin-to-creatinine ratio. * *p* < 0.05 vs. Pre.

Secondary endpoints:

(1) BNP, ANP (Figure 3 and Table 4): Regarding BNP, levels were 78.1 pg/mL (43.3–137.6 pg/mL) during eplerenone treatment; one month after switching to esaxerenone, levels were 81.6 pg/mL (35.4–140.6 pg/mL); 68.2 pg/mL (34.6–122.6 pg/mL) at 3 months, and 62.1 pg/mL (37.7–126.3 pg/mL) at 6 months. BNP levels at 3 and 6 months were significantly lower than baseline (1 month: *p* = 1.00, 3 months: *p* = 0.04, 6 months: *p* = 0.02). Regarding ANP, levels were 69.3 pg/mL (48.7–98.1 pg/mL) during eplerenone treatment, 72.0 pg/mL (42.1–104.0 pg/mL) at 1 month, 69.4 pg/mL (46.0–99.1 pg/mL) at 3 months, and 68.5 pg/mL (46.6–99.1 pg/mL) at 6 months. These differences were not statistically significant (*p* = 0.975).
(2) BUN, s-Cr, eGFR (Table 3): Following the switch from eplerenone to esaxerenone, there was no significant change in BUN (*p* = 0.337), whereas s-Cr decreased significantly and eGFR increased significantly (s-Cr: *p* = 0.017; eGFR: *p* = 0.028).
(3) PRA, Ang II, PAC, cortisol (Figure 4 and Table 3): Regarding PRA, levels increased significantly compared with baseline at both 3 and 6 months after switching from eplerenone to esaxerenone (both *p* < 0.001). Regarding Ang II, there was no significant difference before and after the switch (*p* = 0.35). Regarding PAC, after switching from eplerenone to esaxerenone, levels increased significantly compared to pre-switch values starting 3 months post-switch (3 months: *p* < 0.001, 6 months: *p* = 0.034). There was no significant difference in cortisol levels before and after the switch (*p* = 0.797).
(4) Sodium, potassium (serum, urine), U-Na/K, osm (serum, urine) (Table 3): There were no significant differences in serum sodium or urine sodium before and after the switch (serum: *p* = 0.207, urine: *p* = 0.804). Serum potassium levels rose significantly 3 months or more after the switch (3 months: *p* < 0.001, 6 months: *p* < 0.001), but no patients had serum potassium levels > 5.5 mEq/L. There was no significant difference in urine potassium levels before and after the switch (*p* = 0.559). There was no significant difference in U-Na/K before and after the switch (*p* = 0.759). There was no significant difference in serum osmolality before and after the switch (*p* = 0.905). Urinary osmolality decreased significantly 3 months or more after the switch (3 months: *p* = 0.01, 6 months: *p* = 0.002).
(5) UACR, U-IV collagen, U-β_2_MG, U-LFABP (Figure 5, Table 4): Regarding UACR, levels were 24.9 mg/gCr (9.4–111.5 mg/gCr) before switching to esaxerenone 17.1 mg/gCr (7.4–54.5 mg/gCr) at 3 months, and 14.6 mg/gCr (7.9–42.2 mg/gCr) at 6 months; values at 3 and 6 months were significantly lower than baseline (all *p* < 0.001). There were no significant differences in U-IV collagen, U-β_2_MG, or U-LFABP before and after the switch (U-IV collagen: *p* = 0.232, U-β_2_MG: *p* = 0.195, U-LFABP: *p* = 0.205).

## 4. Discussion

This study investigated the effects of switching from eplerenone, a steroidal MRA, to esaxerenone, a non-steroidal MRA, in patients with chronic heart failure and hypertension. The results showed that although PRA and PAC increased significantly, systolic and diastolic blood pressure, BNP, UACR, serum creatinine and urinary osmolality decreased significantly, whereas eGFR increased significantly. These findings suggest that switching from eplerenone to esaxerenone was associated with favorable changes in cardiorenal biomarkers despite compensatory activation of the RAAS.

Spironolactone and eplerenone, which are conventional MRAs, are steroidal MRAs; the RALES, EPHESUS, and EMPHASIS-HF trials have demonstrated their efficacy in improving the prognosis of patients with heart failure [1,3,4]. In contrast, esaxerenone and finerenone, which were developed in recent years, are non-steroidal MRAs; they have been reported to possess high selectivity for MR and potent MR-blocking effects at the tissue level [5,6,7,8,9,10,11,12,13]. In particular, because non-steroidal MRAs differ from steroidal MRAs in their mode of binding to MR and their tissue distribution, it has been suggested that they may exert stronger anti-inflammatory and anti-fibrotic effects [14]. We previously reported, in a crossover trial of eplerenone and esaxerenone in patients with chronic heart failure and hypertension, that switching to esaxerenone was associated with an increase in PAC together with improvements in BNP, blood pressure, and UACR [11]. The present findings are consistent with those observed in our previous crossover study, suggesting that these physiological responses are also observed following switching in routine clinical practice.

In this study, PRA and PAC increased significantly following the switch from eplerenone to esaxerenone. Although this might initially appear to reflect adverse RAAS activation, it is more appropriately interpreted as a compensatory response to effective MR blockade. Blockade of MR in the distal nephron inhibits sodium reabsorption, leading to increased renin secretion and subsequent stimulation of aldosterone production. In the present study, PAC increased despite no significant change in Ang II, suggesting that factors other than Ang II, including MR blockade-mediated feedback, serum potassium, and local RAAS activity, may contribute to this response. Because esaxerenone has a higher affinity for the MR than eplerenone, the greater increase in PRA and PAC may be consistent with more potent MR blockade. This compensatory increase in PAC should be distinguished from aldosterone breakthrough observed during ACE inhibitor or ARB therapy. Aldosterone breakthrough refers to the re-elevation of aldosterone despite continued RAAS inhibition and has been associated with adverse cardiovascular and renal outcomes [15]. In contrast, the increase in PAC observed in the present study occurred despite continued MR blockade and therefore represents a different pathophysiological phenomenon. Importantly, despite the increase in PAC, the observed physiological changes were associated with reductions in blood pressure, BNP, UACR, serum creatinine, and urinary osmolality, together with an increase in eGFR. These findings suggest that circulating PAC alone does not necessarily reflect the biological activity of aldosterone. Rather, effective blockade of the MR may be more important than circulating PAC levels for cardiorenal protection. These findings are consistent with those of our previous crossover study [11] and support the concept that adequate MR blockade can provide cardiorenal benefits despite compensatory increases in circulating aldosterone.

BNP levels decreased significantly at both 3 and 6 months after the switch. BNP is a well-established biomarker of heart failure that reflects ventricular wall stress, and its decrease is a finding suggestive of reduced cardiac workload. The efficacy of mineralocorticoid receptor antagonists (MRAs) in improving heart failure has been established in the RALES, EPHESUS, and EMPHASIS-HF trials [1,3,4]. Since both systolic and diastolic blood pressure decreased significantly after switching to esaxerenone, it is possible that reduced afterload led to a decrease in left ventricular wall stress, which in turn resulted in the decrease in BNP. Furthermore, MR activation is known to promote myocardial fibrosis, inflammation, oxidative stress, and vascular endothelial dysfunction [16]. Therefore, effective MR blockade may have contributed to improvements in myocardial remodeling and microvascular dysfunction beyond blood pressure reduction alone. The majority of patients in this study had HFpEF, a condition in which elevated blood pressure, left ventricular hypertrophy, myocardial fibrosis, and renal dysfunction are deeply involved in pathogenesis. Consequently, the blood pressure-lowering effect and MR-blocking action of esaxerenone may have been particularly beneficial in HFpEF.

With regard to renal function, this study observed a decrease in s-Cr and an increase in eGFR alongside a reduction in UACR. UACR is not only an indicator of renal impairment but also an important biomarker that reflects cardiovascular events and renal prognosis. Therefore, the reduction in UACR observed in this study may reflect not only improvements in local renal function but also improvements in the cardio-renal relationship. MR activation promotes increased glomerular pressure, podocyte damage, tubulointerstitial inflammation, oxidative stress, and fibrosis, and is involved in the increase in albuminuria [16]. Finerenone significantly reduced renal events in patients with chronic kidney disease in the FIDELIO-DKD trial [5]. Furthermore, regarding esaxerenone, animal studies have reported that PAC induces inflammation, activates MR, and promotes renal fibrosis; it has been shown that esaxerenone suppresses this pathological condition, resulting in a decrease in UACR and suppression of renal fibrosis [6,17]. Clinical cases have also reported a reduction in albuminuria in patients with hypertension and chronic kidney disease [18,19]. A mediation analysis of the ESAX-DN trial reported that part of the renoprotective effect of esaxerenone was independent of blood pressure reduction [19]. The reductions in UACR and improvements in eGFR observed in this study may also reflect the local renal RAAS-inhibitory and anti-fibrotic effects of non-steroidal MRAs. It is generally known that eGFR transiently decreases in the early stages of RAAS inhibitor or MRA therapy due to a reduction in glomerular pressure. However, all patients in this study had been taking eplerenone for more than one year and were already on MR-blocking therapy. Nevertheless, it is an interesting finding that a decrease in serum creatinine and an increase in eGFR were observed after switching to esaxerenone. These findings suggest that the observed renal benefits were not merely due to the initiation of MRA therapy, but rather that esaxerenone, a non-steroidal MRA, may have exerted more potent MR-blocking, anti-inflammatory, and anti-fibrotic effects in renal tissue than eplerenone, a steroidal MRA. Furthermore, while concerns regarding decreased eGFR and hyperkalemia are generally associated with MRA administration, this study did not observe any severe renal dysfunction; rather, eGFR improved. This suggests that switching to esaxerenone can be safely performed from a renal function perspective, provided that patients with an eGFR below 30 mL/min/1.73 m^2^ are excluded and the dosage is carefully adjusted.

Furthermore, this study showed a significant decrease in U-OSM. In this study, serum osmolality remained unchanged, while only urinary osmolality decreased. This finding may reflect changes in water and sodium regulation at the level of the renal collecting ducts rather than systemic osmolality changes. Aldosterone is involved in sodium reabsorption and water retention in the collecting ducts, and MR blockade may alter the ability to concentrate urine. In chronic heart failure, water reabsorption is enhanced due to a decrease in effective circulating plasma volume and activation of the vasopressin system, a key neurohumoral pathway. As a result, urine may become concentrated, leading to elevated U-OSM levels [20]. Therefore, the decrease in U-OSM observed in this study may reflect an improvement in water retention associated with heart failure or a reduction in vasopressin activity. Furthermore, if MR blockade inhibits sodium reabsorption in the collecting ducts, water and electrolyte regulation in the renal tubules may change, potentially affecting urine concentration capacity. Since serum osmolality remained unchanged, the decrease in U-OSM likely reflects changes in renal tubular water and sodium handling associated with improved heart failure status rather than systemic osmotic abnormalities. U-OSM is an indicator that has not been sufficiently examined in general clinical studies of heart failure. However, in this study, U-OSM decreased in parallel with reductions in BNP, blood pressure, and UACR, as well as improvements in eGFR. This finding suggests that switching to esaxerenone may have beneficial effects not only on the cardiorenal axis but also on water homeostasis. Although the clinical significance of U-OSM remains to be fully established, the parallel improvement in BNP, UACR, and eGFR suggests that urinary osmolality may serve as a potential marker of improved cardiorenal water handling following effective MR blockade.

Eighty-eight percent of the patients in this study had HFpEF. In HFpEF, hypertension, myocardial fibrosis, vascular stiffness, and renal dysfunction play key roles in the pathogenesis of the disease. MR activation is closely associated with these pathological conditions, and potent MR blockade with a non-steroidal MRA may theoretically be effective against the pathophysiology of HFpEF. Recently, it was reported in the FINEARTS-HF trial that finerenone, a non-steroidal MRA, significantly reduced heart failure events in patients with HFmrEF and HFpEF [5]. Studies evaluating esaxerenone in patients with heart failure remain limited and are generally small in scale. In a study similar to the present study, esaxerenone was administered to 33 patients with HFpEF and concomitant hypertension, resulting in significant reductions in systolic blood pressure, left ventricular mass index, and BNP, as well as beneficial effects on reverse remodeling [21]. In another study involving 48 patients with chronic heart failure and concomitant hypertension, esaxerenone significantly reduced systolic blood pressure and BNP levels [22]. However, to our knowledge, no previous studies have evaluated patients with heart failure who were switched from another MRA to esaxerenone, nor have any examined changes in the RAAS and renal function in detail. The reductions in BNP and blood pressure, as well as the renal protective effects observed in this study, suggest that esaxerenone may play a beneficial role in patients with HFpEF. Currently, a prospective study (the EAPIAL trial) is underway in Japan comparing esaxerenone with amlodipine in patients with chronic heart failure complicated by hypertension and albuminuria [23], and the results are awaited with interest.

This exploratory study was designed to evaluate physiological changes after switching from a steroidal to a non-steroidal mineralocorticoid receptor antagonist in routine clinical practice rather than to compare treatment efficacy between agents. Therefore, the present findings should be considered hypothesis-generating and warrant confirmation in future randomized controlled studies with clinical outcome measures. Therefore, the observed physiological changes should be interpreted as associations rather than evidence of causal effects. The present findings should be considered hypothesis-generating and warrant confirmation in future randomized controlled studies with clinical outcome measures.

## 5. Conclusions

In patients with chronic heart failure and hypertension, switching from eplerenone to esaxerenone was associated with reductions in blood pressure, BNP, UACR, and urinary osmolality, together with improvements in renal function, despite an increase in PAC. These findings suggest favorable physiological and cardiorenal changes following the switch from a steroidal to a non-steroidal mineralocorticoid receptor antagonist. Although esaxerenone is not currently indicated for heart failure, these findings support further investigation of its potential role in patients with HFpEF and concomitant hypertension. Further randomized studies with clinical outcome measures are warranted.

## 6. Limitations

This study has several limitations. First, this was an exploratory, single-center, open-label, single-arm study without randomization, blinding, or a parallel control group. Therefore, causal relationships between switching from eplerenone to esaxerenone and the observed physiological changes cannot be established with certainty. Second, although this study involved switching from eplerenone to esaxerenone, no washout period was introduced because temporary discontinuation of mineralocorticoid receptor antagonist therapy was considered ethically inappropriate due to the potential risk of worsening heart failure and inadequate blood pressure control. Nevertheless, the absence of a washout period remains an important methodological limitation because it precludes clear separation of the effects of eplerenone withdrawal and esaxerenone initiation. Third, the majority of patients had HFpEF, limiting the generalizability of the findings to patients with HFrEF or HFmrEF. Fourth, the observation period was relatively short at 6 months, and hard clinical outcomes such as hospitalization for heart failure and all-cause mortality were not evaluated. Fifth, all patients had been receiving eplerenone for at least one year before enrollment; therefore, the findings cannot be directly extrapolated to patients who have not previously received MRA therapy. Accordingly, these findings should be interpreted as exploratory and hypothesis-generating and require confirmation in future randomized controlled studies with clinical outcome measures.

## Figures and Tables

**Table 1:** Comparison of the pharmacological and clinical characteristics of eplerenone and esaxerenone.

	Eplerenone	Esaxerenone
Chemical structure	Steroidal MRA	Non-steroidal MRA
Structural formula	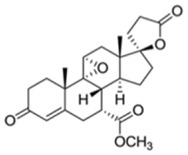	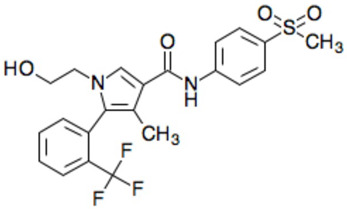
Molecular formula	C_24_H_30_O_6_	C_22_H_21_F_3_N_2_O_4_S
MR selectivity	Selective MR antagonist	Highly selective MR antagonist
Plasma half-life	3–5 h	18–25 h
Approved indication (Japan)	Heart failure and hypertension	Hypertension
Landmark clinical trials	EPHESUS, EMPHASIS-HF	ESAX-HTN, ESAX-DN

MRA: mineralocorticoid receptor antagonist, MR: mineralocorticoid receptor. Data summarized from Arai et al. [8], Wan et al. [9], Duggan [10], and the Japanese prescribing information.

**Table 2:** Baseline patient characteristics.

Number of patients (n)	156
Age (years)	72.9 ± 10.7 (range, 36–88)
Sex, male/female	111/45
Heart failure classification	
HFrEF	11 (7%)
HFmrEF	8 (5%)
HFpEF	137 (88%)
NYHA class (I, II, III)	5, 137, 14
Etiology of heart failure	
Ischemic heart disease	38 (24%)
Valvular heart disease	64 (41%)
Hypertension	29 (19%)
Cardiomyopathy	16 (10%)
Arrhythmias	6 (4%)
Congenital heart disease	3 (2%)
Comorbidities	
Diabetes mellitus	60 (38%)
Dyslipidemia	133 (85%)
CKD (stage G3a)	112 (71%)
CKD (stage G3b)	45 (29%)

HFrEF: heart failure with reduced ejection fraction; HFmrEF: heart failure with mildly-reduced ejection fraction; HFpEF: heart failure with preserved ejection fraction; NYHA: New York Heart Association; CKD: chronic kidney disease; ACE-I: angiotensin-converting enzyme inhibitors; ARB: angiotensin II receptor blocker; ARNI: angiotensin receptor neprilysin inhibitor; SGLT2: sodium–glucose cotransporter 2.

**Table 3:** Changes in parametric variables after switching from eplerenone to esaxerenone.

	Pre	One Month	3 Months	6 Months	*p* Value
Systolic BP (mmHg)	131.5 ± 12.4	121.9 ± 9.9 *	121.8 ± 9.8 *	122.0 ± 9.5 *	<0.001
Diastolic BP (mmHg)	73.0 ± 10.3	69.4 ± 8.5 *	69.5 ± 8.5*	69.0 ± 8.8 *	<0.001
Heart rate (/min)	66.4 ± 8.8	65.4 ± 8.5	66.0 ± 8.9	65.4 ± 8.0	0.215
Cortisol (μg/dL)	11.4 ± 3.2	-	11.4 ± 3.5	11.2 ± 3.8	0.797
Blood urea nitrogen (mg/dL)	19.6 ± 5.9	19.9 ± 6.4	20.1 ± 6.3	20.3 ± 6.2	0.337
Serum creatinine (mg/dL)	1.04 ± 0.25	1.01 ± 0.25 *	1.02 ± 0.26	1.02 ± 0.26 *	0.017
eGFR (mL/min/1.73 m^2^)	53.4 ± 13.7	54.8 ± 14.3 *	54.3 ± 14.6	54.7 ± 14.9 *	0.028
Serum sodium (mEq/L)	140.5 ± 2.7	140.0 ± 5.0	140.4 ± 3.3	140.7 ± 3.1	0.207
Serum potassium (mEq/L)	4.31 ± 0.38	4.31 ± 0.38	4.38 ± 0.39 *	4.41 ± 0.3 9*	<0.001
Urinary sodium (mEq/L)	104.9 ± 48.1	-	104.1 ± 48.3	102.6 ± 48.4	0.804
Urinary potassium (mEq/L)	40.6 ± 21.8	-	47.6 ± 81.1	40.9 ± 42.4	0.559
Urinary Na/K ratio	3.27 ± 2.11	-	3.39 ± 2.16	3.39 ± 2.09	0.759
Serum osmolality (mOsm/kgH_2_O)	289.0 ± 6.4	-	288.8 ± 8.0	289.0 ± 6.9	0.905
Urinary osmolality (mOsm/kgH_2_O)	591.9 ± 238.6	-	537.2 ± 223.6	527.7 ± 210.2	0.002

Values are expressed as mean ± SD. * *p* < 0.05 vs. Pre. BP: blood pressure; eGFR: estimated glomerular filtration rate; Na/K: sodium-to-potassium.

**Table 4:** Non-normally distributed variables.

	Pre	One Month	3 Months	6 Months	*p* Value
ANP (pg/mL)	69.3 (48.7–98.1)	72.0 (42.1–104.0)	69.4 (46.0–106.0)	68.5 (46.6–99.1)	0.975
U-IV collagen (μg/gCr)	5.3 (3.3–7.3)	-	4.5 (3.2–6.7)	4.7 (3.5–7.2)	0.232
U-β_2_MG (μg/L)	159.0 (77.5–374.0)	-	146.2 (60.2–315.1)	150.2 (68.0–338.3)	0.195
U-LFABP (μg/gCr)	3.9 (2.2–6.0)	-	3.6 (2.2–5.8)	3.8 (2.4–5.7)	0.205

ANP: atrial natriuretic peptide; U-IV collagen: urinary type IV collagen; U-β_2_MG: urinary β_2_-microglobulin; U-LFABP: urinary liver-type fatty acid-binding protein.

## Data Availability

The data that support the findings of this study are available on request from the corresponding author. The data are not publicly available because they contain information that could compromise the privacy of research participants.

## References

[1] Pitt B., Zannad F., Remme W.J., Cody R., Castaigne A., Perez A., Palensky J., Wittes J. (1999). The Effect of Spironolactone on Morbidity and Mortality in Patients with Severe Heart Failure. N. Engl. J. Med..

[2] de Souza F., Muxfeldt E., Fiszman R., Salles G. (2010). Efficacy of Spironolactone Therapy in Patients with True Resistant Hypertension. Hypertension.

[3] Pitt B., Remme W., Zannad F., Neaton J., Martinez F., Roniker B., Bittman R., Hurley S., Kleiman J., Gatlin M. (2003). Eplerenone, a Selective Aldosterone Blocker, in Patients with Left Ventricular Dysfunction after Myocardial Infarction. N. Engl. J. Med..

[4] Zannad F., McMurray J.J.V., Krum H., van Veldhuisen D.J., Swedberg K., Shi H., Vincent J., Pocock S.J., Pitt B. (2011). Eplerenone in Patients with Systolic Heart Failure and Mild Symptoms. N. Engl. J. Med..

[5] Bakris G.L., Agarwal R., Anker S.D., Pitt B., Ruilope L.M., Rossing P., Kolkhof P., Nowack C., Schloemer P., Joseph A. (2020). Effect of Finerenone on Chronic Kidney Disease Outcomes in Type 2 Diabetes. N. Engl. J. Med..

[6] Pitt B., Filippatos G., Agarwal R., Anker S.D., Bakris G.L., Rossing P., Joseph A., Kolkhof P., Nowack C., Schloemer P. (2021). Cardiovascular Events with Finerenone in Kidney Disease and Type 2 Diabetes. N. Engl. J. Med..

[7] Solomon S.D., McMurray J.J.V., Vaduganathan M., Claggett B., Jhund P.S., Desai A.S., Henderson A.D., Lam C.S.P., Pitt B., Senni M. (2024). Finerenone in Heart Failure with Mildly Reduced or Preserved Ejection Fraction. N. Engl. J. Med..

[8] Arai K., Tsuruoka H., Homma T. (2015). CS-3150, a novel non-steroidal mineralocorticoid receptor antagonist, prevents hypertension and cardiorenal injury in dahl salt-sensitive hypertensive rats. Eur. J. Pharmacol..

[9] Wan N., Rahman A., Nishiyama A. (2021). Esaxerenone, a novel nonsteroidal mineralocorticoid receptor blocker (MRB) in hypertension and chronic kidney disease. J. Hum. Hypertens..

[10] Duggan S. (2019). Esaxerenone: First global approval. Drugs.

[11] Sezai A., Abe M., Maruyama T., Taoka M., Sekino H., Tanaka M. (2025). A Prospective Crossover Clinical Trial of Esaxerenone and Eplerenone in Patients with Chronic Heart Failure Complicated by Hypertension. Life.

[12] Ito S., Itoh H., Rakugi H., Okuda Y., Yoshimura M., Yamakawa S. (2020). Double-blind randomized phase 3 study comparing esaxerenone (CS-3150) and eplerenone in patients with essential hypertension (ESAX-HTN Study). Hypertension.

[13] Kario H., Ito S., Itoh H., Rakugi H., Okuda Y., Yoshimura M., Yamakawa S. (2021). Effect of the nonsteroidal mineralocorticoid receptor blocker, esaxerenone, on nocturnal hypertension: A post hoc analysis of the ESAX-HTN study. Am. J. Hypertens..

[14] Jaisser F., Farman N. (2016). Emerging Roles of the Mineralocorticoid Receptor in Pathology. Nat. Rev. Endocrinol..

[15] Struthers A.D. (1995). Aldosterone escape during ACE inhibitor therapy in chronic heart failure. Eur. Heart J..

[16] Struthers A.D., MacDonald T.M. (2004). Review of aldosterone-and angiotensin II-induced target organ damage and prevention. Cardiovasc. Res..

[17] Qiang P., Hao J., Yang F., Han Y., Chang Y., Xian Y., Xiong Y., Gao X., Liang L., Shimosawa T. (2022). Esaxerenone inhibits the macrophage-to-myofibroblast transition through mineralocorticoid receptor/TGF-β1 pathway in mice induced with aldosterone. Front. Immunol..

[18] Ito S., Kashihara N., Shikata K., Nangaku M., Wada T., Okuda Y., Sawanobori T. (2021). Efficacy and safety of esaxerenone (CS-3150) in Japanese patients with type 2 diabetes and macroalbuminuria: A multicenter, single-arm, open-label phase III study. Clin. Exp. Nephrol..

[19] Okuda Y., Ito S., Kashihara N., Shikata K., Nangaku M., Wada T., Sawanobori T., Taguri M. (2023). The renoprotective effect of esaxerenone independent of blood pressure lowering: A post hoc mediation analysis of the ESAX-DN trial. Hypertens. Res..

[20] Sanghi P., Uretsky B.F., Schwarz E.R. (2005). Vasopressin antagonism: A future treatment option in heart failure. Eur. Heart J..

[21] Imamura T., Oshima A., Narang N., Kinugawa K. (2021). Implication of mineralocorticoid receptor antagonist esaxerenone in patients with heart failure with preserved ejection fraction. Circ. Rep..

[22] Naruke T., Maemura K., Oki T., Yazaki M., Fujita T., Ikeda Y., Nabeta T., Ishii S., Minami Y., Fukaya H. (2021). Efficacy and safety of esaxerenone in patients with hypertension and concomitant heart failure. Hypertens. Res..

[23] Suna S., Matsumoto Y., Niki K., Asanuma H., Higuchi Y., Yasumura Y., Kawarabayashi T., Izumi M., Okuhara Y., Hasegawa S. (2025). Rationale and Design of the ESPIAL Trial—A Prospective, Randomized, Exploratory Study to Evaluate the Effect of Esaxerenone on Reduction of Urinary Albumin to Creatinine Ratio in Hypertensive Patients Concomitant with Heart Failure and Albuminuria. Circ. Rep..

